# Role of microperimetry in evaluating disease progression in age-related macular degeneration: a scoping review

**DOI:** 10.1007/s10792-021-02170-9

**Published:** 2022-01-07

**Authors:** Gopinath Madheswaran, Pinaz Nasim, Shonraj Ballae Ganeshrao, Rajiv Raman, Ramesh S. Ve

**Affiliations:** 1 Department of Optometry, Manipal College of Health Professions, Manipal Academy of Higher Education, Manipal, Karnataka India; 2 Shri Bhagwan Mahavir Vitreoretinal Services, Sankara Nethralaya, Chennai, Tamilnadu India

**Keywords:** Age-related macular degeneration, Mesopic microperimetry, Microperimeter, Fundus controlled perimetry, Scotopic microperimetry, Scoping review

## Abstract

**Purpose:**

Recent research has found variable evidence on the role of mesopic and dark-adapted scotopic microperimetry assessment in age-related macular degeneration. This scoping review summarises how mesopic and scotopic microperimetry can be used to assess disease progression in age-related macular degeneration and identifies gaps in the literature.

**Methods:**

A population, concept, and context approach was used to develop the search strategy. Ovid MEDLINE, EMBASE, Cochrane Library, PubMed, CINAHL Plus, Web of Science, and SCOPUS databases were used to conduct the literature search. The key search terms used in the databases were age-related macular degeneration and microperimetry.

**Results:**

Twelve studies were eligible and included in the review. All the studies (*n* = 12) were conducted in European countries [Germany (9), Italy (2), and the United Kingdom (1)]. The mesopic and scotopic sensitivities were measured using the Nidek scotopic microperimeter (MP1-S) (*n* = 6), scotopic Macular Integrity Assessment device (S-MAIA) (*n* = 5), and both MP1-s and S MAIA (*n* = 1). 83.3% (*n* = 10) studied (cross-sectional design) on mesopic, scotopic microperimetry and found reduced rod (scotopic) photoreceptors sensitivities compared to cone (mesopic) photoreceptors sensitivities in patients with small and reticular pseudodrusen despite having good visual acuity. Only 16.7% (*n* = 2) of studies followed participants with reticular drusen/large drusen for three years (longitudinal design) and found reduced scotopic over mesopic sensitivity at baseline and localized mesopic with profound scotopic sensitivity loss during follow-ups.

**Conclusion:**

Scotopic sensitivity is a better functional indicator than mesopic sensitivity to understand early and intermediate age-related macular degeneration progression. The evidence from longitudinal studies is debatable due to the limited stimuli range of existing microperimeters, smaller sample size, and lost follow-ups.

**Supplementary Information:**

The online version contains supplementary material available at 10.1007/s10792-021-02170-9.

## Introduction

Age-related macular degeneration (AMD) is the third most leading cause of irreversible blindness in the elderly population [1]. The global prevalence of AMD is expected to increase to 10 million by 2040, from 1.8 million in 2020 [2, 3]. AMD is classified based on clinical and imaging findings [4, 5]. Early and intermediate AMD are characterized by the presence of drusen of different sizes and quantities, as well as pigmentary abnormalities [5]. The signs of advanced AMD include choroidal neovascularisation and or geographic atrophy (GA). Depending on the presence or absence of neovascularization, AMD may also be divided into two groups: dry or non-neovascular AMD (or GA) and wet or neovascular AMD (nAMD). Visual impairment in early and intermediate AMD is less severe than in advanced AMD [5, 6]. Wet AMD (nAMD) causes a sudden loss of vision, whereas dry AMD (GA) leads to a gradual, progressive loss of vision [7]. The progression from early or intermediate to advanced AMD is evaluated by comparing structural damage of the retina and functional change in vision [8]. The gold-standard treatment for nAMD is the anti-vascular endothelial growth factor, which has improved prognosis. However, no specific treatment is available for dry AMD [6, 9]. Management of early and intermediate AMD involves closer follow-up examinations of both structural and functional changes.

Microperimetry or fundus-oriented perimetry plays a vital role in testing the foveal sensitivity in AMD [10, 11]. Recent advances in microperimeter include mesopic, scotopic sensitivity tests that provide better diagnostic and prognostic information for AMD [12]. Mesopic and scotopic sensitivity, which measures the cone and rod functions, are estimated by modifying the background illumination of the microperimeter [13, 14].

Commercially available microperimeters such as the Nidek microperimeter (MP1) (NIDEK Technologies, Padova, Italy) was modified by adding the neutral density (ND) filter (MP1-S) [15] to extend the range of stimulus intensity [13, 16]. MP1-S was limited due to its ceiling effects, filter selection based on AMD severity, longer test duration, and poor fixation [16–18]. The latest Nidek MP version 3, which includes scotopic testing [19], claims to have overcome the MP1-S's limitations. However, no studies have evaluated AMD patients' mesopic and scotopic sensitivities using the latest Nidek MP Version 3. Macular Integrity Assessment (MAIA) microperimeter (CenterVue, Padova, Italy), with scotopic testing (S-MAIA) device, was designed to perform the mesopic and dark-adapted scotopic tests using two colored (cyan and red) light-emitting diodes [20]. The use of two-colored stimuli helps determine distinct sensitivity loss of cone and rod photoreceptor’s cells [14, 21–23]. The latest version of S–MAIA features a rapid test protocol and an increased range of stimuli intensity (0–36 decibel), reducing ceiling effects [24].

Recent evidence suggests that scotopic sensitivity is a good functional indicator to assess progression in AMD [13, 23]. The rod photoreceptors (scotopic sensitivity) functions are significantly affected in early and intermediate AMD without affecting the best-corrected visual acuity (BCVA) [8]. Studies reported compared to normal, mesopic and scotopic sensitivity reduction was observed in AMD [14, 16, 21, 23, 25]. A study by Montesano et al. [25] reported that mesopic sensitivity was well correlated with morphological changes than scotopic sensitivity. However, other studies have suggested scotopic sensitivity as a better functional indicator [13, 16, 23, 26, 27] for detecting AMD progression, associated with an increased risk of blindness.

Scoping reviews offer an effective approach for identifying and mapping the available evidence, providing critical concepts and key features in a specific study area [28]. The key objectives of this scoping review are: (a) To understand the role of mesopic and scotopic microperimetry sensitivities in AMD, (b) To understand if mesopic and scotopic sensitivities help identify patients progressing from early or intermediate AMD, (c) To map the research carried out in this field systematically, and (d) To find gaps in the existing literature.

## Methodology

The scoping review protocol was registered (OSF registries) [29] and followed the elaborated methodological framework for scoping reviews by Levac et al. [30, 31]. The steps followed were: (1) identification of the research objectives; (2) identification of relevant studies; (3) screening and selection of studies; (4) charting the data; and (5) collating, summarizing, and reporting the results.

### Identification of the research objective

The research objectives discussed in the introduction were formulated. A search strategy was developed using the population, concept, and context (PCC) approach [32].

- *Population* People with age-related macular degeneration,
- *Concept* Mesopic and scotopic microperimetry to evaluate disease progression,
- *Context* No limit to gender, race, and geographic location.

### Identification of relevant studies

Electronic records were searched using Ovid MEDLINE, EMBASE, Cochrane Library, PubMed, CINAHLPlus, Web of Science, and SCOPUS on 10th February 2021. Search terms used in the database were “age-related macular degeneration”, “scotopic microperimetry”, “mesopic microperimetry”, “fundus controlled perimetry”, and the related terms that are used provided in online resource 1. In addition, other sources such as theses, dissertations, and grey literature were searched. There were no gender or race restrictions.

### Screening and selection of studies

Titles and abstracts of the identified articles were screened based on the following inclusion and exclusion criteria. This review included subjects with defined stages of age-related macular degeneration by Ferris et al. [5] or Age-Related Eye Disease Study [4]; use of microperimetry or mesopic and scotopic microperimetry, or dark-adapted perimetry. The review excluded study subjects with any other retinal pathologies; studies that do not define AMDs category; studies that do not use mesopic and scotopic microperimetry; studies that do not provide a clear concept and methodology; studies not published full text in scientific journals, English language; conference abstracts.

Studies were imported into Mendeley Desktop [33] to remove the duplicates. The studies were exported to Microsoft Excel 2013 for management and selection based on titles and abstracts by two authors. Studies were selected independently and blind to the decision of the other authors. Studies that failed to meet the criteria were removed. These process differences were resolved by a third reviewer, leading to an agreement. Later, the full text of eligible studies was acquired and read. The details for the exclusion were noted. Also, cited references were searched from the extracted articles with the inclusion and exclusion criteria. The study selection process was documented in a flow chart, according to the Preferred Items guidelines for Systematic Review and Meta-Analysis Reports (PRISMA-ScR) for scoping reviews [34].

### Charting the data

A predetermined form was used to chart the data from the selected studies using Microsoft Excel. Data extracted from the selected studies included study demographics (author, year of publication), methodology (purpose, sample size, and population), results, and key findings. The data extraction, charting were performed independently by two authors and reviewed by a third author.

### Collating, summarizing, and reporting the results

The important information was categorized and tabulated from the included studies by two authors, verified, and approved by a third author. Finally, the current gaps in research on mesopic and scotopic microperimetry in age-related macular degeneration were listed.

## Results

**Fig. 1:**
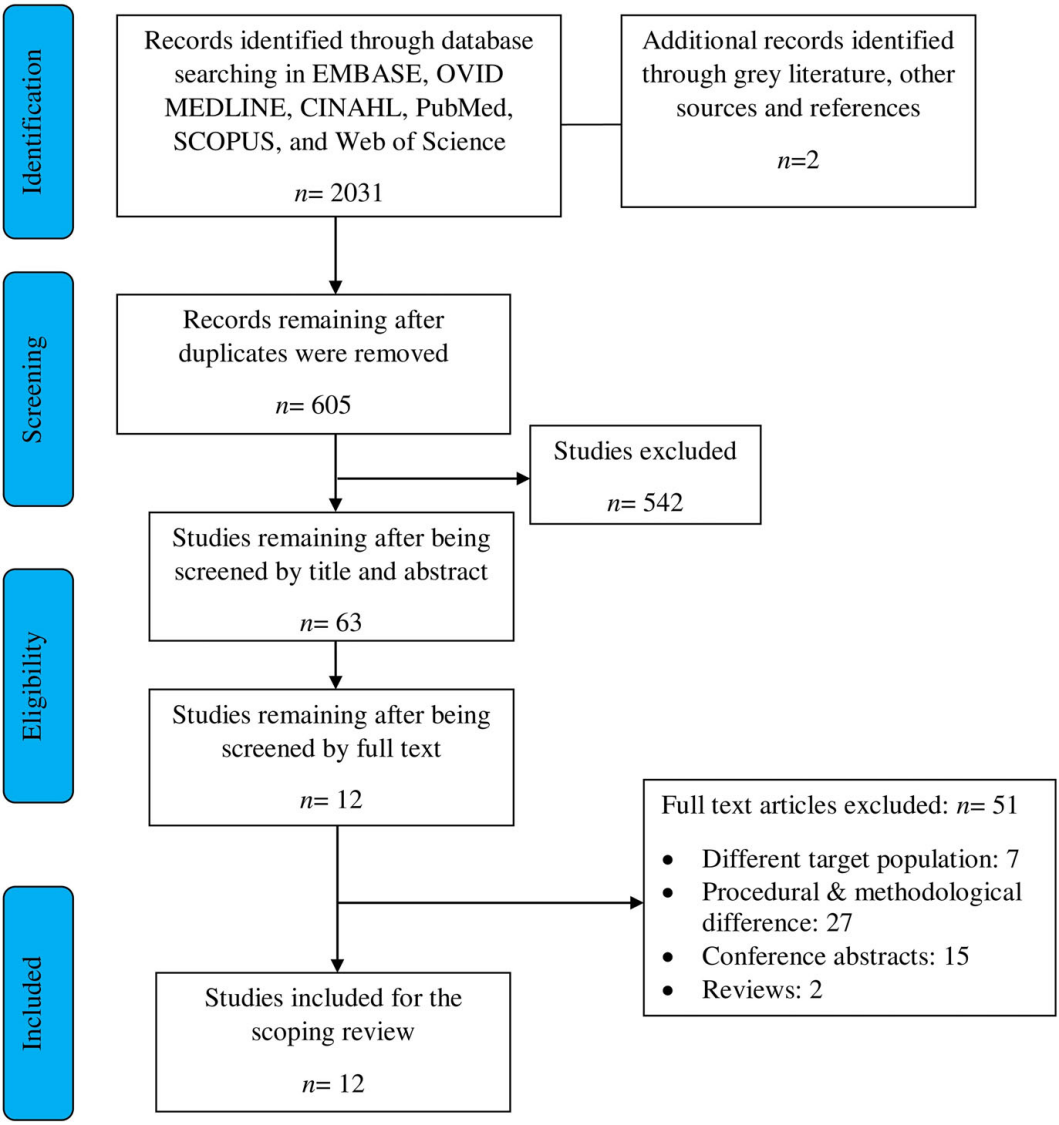
Flow chart on literature search and study selection

The database search identified a total of 2031 articles and two dissertations. (Fig. 1). The remaining records after duplicates removal were 605. Those articles were subjected to titles and abstract screening, and 542 were excluded. The remaining 63 articles were obtained as full text and read, of which 51 articles were excluded. The major reasons for exclusion were the procedural and methodological differences (27), conference abstracts (15), diverse target population (Stargardt’s disease, mixed retinal diseases, and healthy normal) (7), and reviews (2). Two dissertations [35, 36] were obtained as grey literature in a web search; however, they did not meet our criteria and were excluded. Of the 51 articles excluded, 19 articles used microperimetry as a tool for assessing AMD. However, these studies did not measure scotopic or mesopic sensitivities and were excluded. The detailed list for exclusion is provided in online resource 2. A total of twelve articles were included in the final review.

Most of the included studies were done in Germany *n* = 9 (75%), followed by Italy *n* = 2 (16.7%) and United Kingdom *n* = 1 (8.3%). Of the included studies, *n* = 10 (83.3%) followed cross-sectional study design [13, 14, 16, 21–23, 25–27, 37], and *n* = 2 (16.7%) followed longitudinal analysis [38, 39]. Six studies [13, 16, 26, 37–39] included a modified version of microperimeter (MP1-S) [15], five studies used scotopic MAIA (S-MAIA) microperimeter [21–23, 25, 27] and, one study [14] used both MP1-S and S MAIA microperimeters to measure scotopic, mesopic sensitivities. Table 1 summarizes the findings of cross-sectional studies, including the number of participants, mean age, BCVA, mesopic, scotopic sensitivities, and differences in sensitivities between cases (AMD) controls. Mesopic sensitivity is not different between early AMDs [(small drusen and reticular pseudodrusen (RPD)] and healthy controls (*p* > 0.05), as shown in Table 1. However, there is a statistically significant difference in both mesopic and scotopic sensitivities between intermediate AMD (large drusen) and healthy controls (*p* < 0.05).

**Table 1:** Study details, no of participants, mean age, BCVA, mesopic, scotopic sensitivities, and differences in sensitivities among cases and controls from cross-sectional studies

Study	Participants (no of eyes)	Age (years)	Mean BCVA (log MAR)	Mesopic sensitivity mean (SD) (dB)	Scotopic sensitivity mean (SD) (dB)	Difference in mesopic sensitivity (cases—controls) (*p*-value)	Difference in scotopic sensitivity (cases—controls) (*p-*value)
Nebbioso et al. [13], Italy (MP-1S)	Hard drusen (*n* = 12)	66.3 (3.4)	0.0	19.07 (0.21)	5.20 (1.2)	1.9 dB (*p* > 0.05)	2.5 dB (*p* < 0.003)*
	Healthy controls (*n* = 12)	67.1 (7.5)	0.0	19.17 (0.99)	7.70 (0.2)		
Steinberg et al. 2015 [16], Germany (MP-1S)	Early/iAMD (*n* = 18)	74.7 (7.1)	0.0	17.2 (3.61)	13.5 (2.9)	0.9 dB (*p* = 0.03)	4.8 dB (*p* ≤ 0.001)*
	Late AMD (*n* = 4)	74.7 (7.1)	0.2	18.1 (2.40)	18.3 (3.1)		
Steinberg et al. [34], Germany (MP-1S)	RPD (*n* = 20)	75.8 (8.5)	0.1	17.2 (2.50)	12.8 (3.3)	1.2 dB (*p* = 0.01)	5.4 dB (*p* < 0.001)*
	Healthy controls (*n* = 20)	75.5 (10.1)	0.0	18.4 (2.50)	18.2 (2.2)		
Sassmannshausen et al. [25], Germany (MP-1S)	Intermediate AMD (*n* = 35)	70.9 (8.2)	0.1	16.9 (3.00)	14.0 (3.7)	–	–
	Healthy controls (*n* = 29)	75.3 (5.2)	0.0	NR	NR		
Pfau et al. [22], Germany (S- MAIA)	Drusen (*n* = 24)	69.4 (12.6)	0.07	24.9 (2.40)	10.1 (3.0) cyan 12.2 (2.4) red	–	–
	Healthy controls (*n* = 20)	61.7 (12.4)	0.0	NR	NR		
Welker et al. [21], Germany (S- MAIA)	Intermediate AMD (*n* = 23)	67.3 (8.2)	0.4	23.01 (3.30)	19.92 (4.06)	2.62 dB (*p* < 0.01)*	2.49 dB (*p* < 0.01)*
	Healthy controls (*n* = 29)	61.3 (5.2)	0.0	25.63 (2.29)	22.41 (2.54)		
Corvi et al. [14], Italy (MP1-S, S MAIA)	Drusen (*n* = 15)	72.8 (7.1)	0.0	25.44 (4.3)	13.25 (5.5)	2.07 dB (*p* < 0.001)*	4.99 dB (*p* < 0.001)*
	RPD (*n* = 14)	78.2 (7.2)	0.0	23.37 (4.2)	8.26 (5.4)		
von der Emde et al. [27], Germany (S- MAIA)	Neovascular AMD (*n* = 50)	76.1 (7.6)	0.38	NR	NR (cyan) NR (red)	–	2.63 dB (*p* < 0.001)*
	Healthy controls (*n* = 29)	55.9 (16.9)	0.03	NR	NR		
Pondorfer et al. [23], Germany (S- MAIA)	Intermediate AMD (n = 38)	69.1 (7.5)	0.2	23.1 (1.80)	20.0 (2.7)	2.8 dB (*p* < 0.01)*	2.5 dB (*p* < 0.01)*
	Healthy controls (*n* = 24)	61.7 (6.1)	0.0	25.9 (1.60)	22.5 (1.5)		
Montesano et al. [25], United Kingdom (S- MAIA)	Drusen (*n* = 43)	72 (12)	0.1	24.32 (2.48)	10.43 (2.9) cyan 12.53 (3.6) red	1.16 dB (*p* > 0.05)	NR
	Healthy controls (*n* = 56)	62 (5)	0.0	25.48 (1.62)	11.74 (2.0) cyan 13.23 (1.7) red		

*Indicate statistically significant

*SD* standard deviation, *BCVA* best-corrected visual acuity, *dB* decibels, *RPD* reticular pseudo drusen

Only two studies [38, 39] longitudinally followed up patients with RPD and large drusen over three years. At baseline, there was a significant difference in mesopic to scotopic sensitivity between healthy controls and RPD cases [38] (*p* < 0.001). In addition, eyes with large drusen had lower mesopic and scotopic sensitivity at baseline than control participants [39] (*p* < 0.001). Table 2 shows study details, number of participants, mean age, BCVA, mesopic, scotopic sensitivities, retinal thickness, and differences in AMD cohorts at 12, 24, and 36 months from longitudinal studies. From Table 2, it is evident that in both cases of RPD and large drusen, loss of mesopic and scotopic sensitivities are significantly higher during follow-up visits (*p* < 0.001). In addition, in the second and third follow-ups, there was a significant decrease in total retinal thickness at the level of drusen (*p* < 0.001).

**Table 2:** Study details, number of participants, mean age, BCVA, mesopic, scotopic sensitivities, retinal thickness, and differences in AMD cohorts at 12, 24, and 36 months from longitudinal studies

Study variables	Sassmannshausen et al. [36], Germany (MP1-S)	Baseline to follow-up difference (*p-*value)	Study variables	Sassmannshausen et al. [37], Germany (MP1-S)	Baseline to follow-up difference (*p*-value)
Participants (no of eyes)	Reticular pseudo drusen		Participants (no of eyes)	Intermediate AMD	
Baseline	*n* = 35		Baseline	*n* = 59	
Follow-up 1 at 12 months	*n* = 20		Follow-up 1 at 12 months	*n* = 38	
Follow-up 2 at 24 months	*n* = 12		Follow-up 2 at 24 months	*n* = 25	
Follow-up 3 at 36 months	*n *= 11		Follow-up 3 at 36 months	*n* = 14	
Age (years)	72.1 (9.6)		Age (years)	71.72 (8.9)	
Mean BCVA (logMAR)			Mean BCVA (logMAR)		
Baseline	0.1 IQR [0.0–0.2]		Baseline	0.1	
Follow-up 1 at 12 months	0.1 IQR [0.1–0.2]	–	Follow-up 1 at 12 months	NR	–
Follow-up 2 at 24 months	0.2 IQR [0.0–0.3]	–	Follow-up 2 at 24 months		–
Follow-up 3 at 36 months	0.2 IQR [0.1–0.4]	–	Follow-up 3 at 36 months		–
Mean mesopic sensitivity at RPD (dB) (mean SD)			Mean mesopic sensitivity (dB) (mean SD)		
Base line	17.49 (2.34)		Base line		0.35 dB loss/reduction per year (*p* < 0.001)*
Follow-up 1 at 12 months	14.54 (2.14)	2.95 (*p* < 0.001)*	Follow-up 1 at 12 months	NR	
Follow-up 2 at 24 months	11.38 (2.13)	6.11 (*p* < 0.001)*	Follow-up 2 at 24 months		
Follow-up 3 at 36 months	10.36 (3.71)	7.13 (*p* < 0.001)*	Follow-up 3 at 36 months		
Mean scotopic sensitivity at RPD (dB) (mean SD)			Mean scotopic sensitivity (dB) (mean SD)		
Baseline	14.03 (2.97)		Baseline		0.20 dB gain/increase per year (*p* < 0.001)*
Follow-up 1 at 12 months	13.54 (2.54)	0.49 (*p* = 0.116)	Follow-up 1 at 12 months	NR	
Follow-up 2 at 24 months	9.97 (2.89)	4.12 (*p* < 0.001)*	Follow-up 2 at 24 months		
Follow-up 3 at 36 months	9.70 (3.71)	4.33 (*p* < 0.001)*	Follow-up 3 at 36 months		
Total retinal thickness (µm) (mean SD)			Total retinal thickness (µm) (mean SD)		
Baseline	302.97 (17.31)		Baseline		Increasing thickness of RPE by 0.51 µm/year (*p* < 0.001)*
Follow-up 1 at 12 months	302.03 (18.02)	0.94 (*p* > 0.05)	Follow-up 1 at 12 months	NR	
Follow-up 2 at 24 months	296.01 (23.23)	6.96 (*p* < 0.001)*	Follow-up 2 at 24 months		
Follow-up 3 at 36 months	292.60 (19.83)	10.37 (*p* < 0.001)*	Follow-up 3 at 36 months		

*Indicate statistically significant

*IQR* interquartile range, *SD* standard deviation, *BCVA* best-corrected visual acuity, *dB* decibels, *µm* micrometer, *NR* not reported, *SD-OCT* retinal thickness measured using spectral-domain optical coherence tomography, *RPE* retinal pigment epithelium

Table 3 compares the mesopic and scotopic sensitivity testing protocols between MP1-S and S-MAIA.

**Table 3:** Mesopic and scotopic sensitivity testing protocols using MP1-S and S-MAIA

Study	Device	Test	Target	Grid	Background	Stimuli	Strategy	Room illumination	Adaptation
Nebbiolo et al. [13]	MP1-S	Mesopic	Single cross at central 2°	Central 6°, 39 points	1.27 cd/m^2^	Goldmann III, 200 ms	FT, 4–2 staircase	Dark room (< 0.01 lx)	Light adapted
	MP1-S	Scotopic	Circle at central 2°	Central 6°, 39 points	0.0032 cd/m^2^	Goldmann V, 200 ms	FT, 4–2 staircase	Dark room (< 0.01 lx)	30 min dark adapted
Steinberg et al. [16, 35], Sassmannshausen et al. [26, 36, 37]	MP1-S	Mesopic	Ring with 3° radius	Central 20°, 56 points	1.27 cd/m^2^	Goldmann III, 200 ms	FT, 4–2 staircase	Darkroom (< 0.01 lx)	Light adapted
	MP1-S	Scotopic	Ring with 3° radius	Central 20°, 56 points	0.0032 cd/m^2^	Goldmann V, 200 ms	FT, 4–2 staircase	Dark room (< 0.01 lx)	20–30 min dark-adapted
Pfau et al. [22]	S- MAIA	Mesopic	Red ring of 1° diameter	Central 18°, 49 points	1.27 cd/m^2^	Goldmann III, 200 ms, 400- 800 nm	FT, 4–2 staircase	Dark room (< 0.01 lx)	Light adapted
	S- MAIA	Scotopic	Red ring of 1° diameter	Central 14°, 49 points	< 0.0001 cd/m^2^	Goldmann III, 200 ms, 507 nm and 627 nm	FT, 2–1 staircase	Darkroom (< 0.01 lx)	30 min dark-adapted
Welker et al. [21], Pondorfer et al. [23]	S- MAIA	Mesopic	Red ring of 1° diameter	Customized, 33 points	1.27 cd/m^2^	Goldmann III, 200 ms, 850 nm	FT, 4–2 staircase	Dark room (< 0.01 lx)	Light-adapted
	S- MAIA	Scotopic	Red ring of 1° diameter	Customized, 33 points	< 0.0001 cd/m^2^	Goldmann III, 200 ms, 627 nm	FT, 4–2 staircase	Dark room (< 0.01 lx)	30 min dark-adapted
von der Emde et al. [27]	S- MAIA	Mesopic	Not reported	Central 18°, 61 points	1.27 cd/m^2^	Goldmann III, 200 ms, 400- 800 nm	FT, 4–2 staircase	Dark room (< 0.01 lx)	Light adapted
	S- MAIA	Scotopic	Not reported	Central 18°, 61 points	< 0.0001 cd/m^2^	Goldmann III, 200 ms, 507 nm and 627 nm	FT, 4–2 staircase	Dark room (< 0.01 lx)	30 min dark adapted
Corvi et al. [14]	MP1-S	Mesopic	Not reported	Customized, 16 points	1.27 cd/m^2^	Goldmann III, 200 ms	FT, 4–2 staircase	Dark room (< 0.01 lx)	Light adapted
	S- MAIA	Scotopic	Not reported	Customized, 16 points	0.0032 cd m^2^	Goldmann V, 200 ms	FT, 4–2 staircase	Dark room (< 0.01 lx)	35 min dark adapted
Montesano et al. [25]	S- MAIA	Mesopic	Ring of 1° diameter	Customized, 44 points	1.27 cd/m^2^	Goldmann III, 200 ms,	FT, 4–2 staircase	Dark room (< 0.01 lx)	Light adapted
	S- MAIA	Scotopic	Ring of 1° diameter	Customized, 44 points	< 0.0001 cd/m^2^	Goldmann III, 200 ms, 505 nm and 627 nm	FT, 2–1 staircase	Dark room (< 0.01 lx)	30 min dark adapted

*MP1-S* Nidek scotopic microperimeter, *S-MAIA* Scotopic Macular Integrity Assessment, *cd/m*^*2*^ indicates candela per square meter, *ms* indicates millisecond, *FT* indicates full threshold

## Discussion

This scoping review summarises the important studies on scotopic and mesopic microperimetry in the results section. The discussion has been categorized as follows: (1) Role of mesopic and scotopic microperimetry in AMD, and (2) Mesopic and scotopic microperimetry in detecting the progression of AMD.

### Role of mesopic and scotopic microperimetry in AMD

Central visual acuity is preserved in the early stages of AMD [40]. Despite having good visual acuity (≥ 6/9) in most participants, studies by Nebbioso et al. [13] and Steinberg et al. [16] found a significant reduction in scotopic sensitivity at the drusen and RPD locations. These findings correlate well with patient-reported symptoms of difficulties in dark-adapted environments. In addition, participants with RPD showed a localized decrease in scotopic sensitivity over mesopic compared to those with drusen [16, 17]. Steinberg et al. [16, 37] and Sabmannshausen et al. [26] also showed a difference in retinal thickness between RPD and drusen. These findings are in line with previous studies [41, 42].

In dark-adapted two-colour microperimetry, reduced scotopic cyan sensitivity was observed, suggesting that rod photoreceptor cells are more impaired than cones in early AMD [22]. Welker et al. [21] reported reduced mesopic and scotopic (cyan, red) sensitivities in intermediate AMD due to moderate visual impairment [43] (≤ 6/18). von der Emde et al. [27] observed a similar sensitivity reduction in neovascular AMD. In contrast with the previous study methods [13, 16, 21, 26, 27, 37], Corvi et al. [14] used two instruments to obtain mesopic (S -MAIA) and scotopic (MP1-S) sensitivities. Corvi et al. [14] found that RPD had reduced scotopic and mesopic functions and better performance in mesopic tests with fewer test points and shorter test duration when using S-MAIA.

Pondorfer et al. [23] also reported reduced mesopic, dark-adapted sensitivities in intermediate AMD. They suggested that mesopic function served better than scotopic functions and concluded that it might overperform mesopic for clinical studies [23]. In comparison with Pfau et al. [22], the notable differences were the increased stimuli intensity, reduced ceiling effect, and participants included with intermediate AMD [23]. Montesano et al. [25] observed mesopic and dark-adapted scotopic (cyan and red) reduction in AMD. Furthermore, mesopic microperimetry outperformed scotopic microperimetry in terms of predicting progression through morphological changes [25].

### Mesopic and scotopic microperimetry in detecting the progression of AMD

Longitudinal studies suggest that comparing the structural and functional changes is a key to progression analysis in AMD [44, 45]. Sassmannshausen et al. [38] observed that participants with RPD had reduced scotopic sensitivity over mesopic at baseline than controls, as well as an equal loss of mesopic and scotopic sensitivity over time (3 years) in RPD. A significant change in the drusen size was the primary reason for reduced mesopic and scotopic sensitivities. Sassmannshausen et al. [39] reported localized mesopic and profound scotopic sensitivity loss in patients with intermediate AMD compared to baseline. There was a significant loss of mesopic sensitivity in follow-ups, but an increase in scotopic sensitivity was observed in patients with large drusen. However, both studies were limited, with a loss in the follow-up of small samples.

In the seven of twelve articles included, only one eye of the patient was examined and included for analysis. However, five articles [16, 26, 37–39] had included both eyes and did not perform between eyes statistical correction to include both eyes. There is a significant between-eye correlation for dry AMD, and statistical correction must be used if both eyes are included in the analysis [46].

## Strengths and limitations

Scoping review methodology allowed for the collection of diverse literature with a comprehensive search using seven databases. However, the review also had several limitations. First, the subjective nature of the article selection due to the scoping review methodology to collect all evidence that might contribute to the study aim. Second, only studies that used commercially available microperimeters were considered; studies that used modified versions or were still in the development stage were not included. Finally, abstracts from conferences were excluded; however, many abstracts were later published in full text. Despite these limitations, this scoping review followed the rigor methodology advocated by Arksey and O’Malley [30], Levac et al. [31], and detailed reporting guidelines and their extensions by Tricco et al. [34].

## Recommendations

### Key findings

Mesopic microperimetry takes less time and is easy to perform, though it does not measure the photoreceptors in the retina compared to scotopic microperimetry. Scotopic microperimetry is more sensitive to detect and differentiate the rod-cone cells changes but requires prior dark adaptation. Patients with early AMD with good visual acuity are more tolerant of scotopic testing than intermediate AMD. Scotopic and mesopic testing is more sensitive in early AMD than intermediate AMD/ advanced AMD. Both MP1-S and S-MAIA are suitable for monitoring progression; however, S-MAIA has the advantage of fast test protocols with a dynamic stimuli range, which helps overcome the longer test duration in MP1-S for scotopic testing filter selection.

### Clinical implications

Microperimeter use in clinical practice will aid in monitoring AMD progression. Studies suggest that structural and functional assessments such as a change in retinal thickness using optical coherence tomography, best-corrected visual acuity, and the foveal sensitivity (scotopic and mesopic microperimetry) should be combined to monitor AMD progression.

## Research

Future research should focus on recruiting a cohort of dry and wet AMD patients at various stages and following them up regularly to understand mesopic and scotopic microperimetry in assessing disease progression. The significant limitation reported in the longitudinal studies was the total time to perform the test and the lack of dynamic stimuli. Software update and developing customized grids will help ease these limitations. Because most published studies were focused on the European population, similar or conflicting results might be expected if done on a diverse study population.

## Conclusion

In conclusion, compared to structural changes, a reduced scotopic function over mesopic at baseline may be used as a functional biomarker to monitor early (drusen, reticular pseudodrusen) and intermediate AMD progression. Longitudinal follow-up studies, on the other hand, are required for more substantial evidence.

## Supplementary Information

Below is the link to the electronic supplementary material.

Supplementary file1 (DOCX 15 KB)

Supplementary file2 (DOCX 15 KB)

## Data Availability

Only published articles were acquired for this scoping review. Data supporting this review is publicly available.

## References

[1] Adelson JD, Bourne RRAA, Briant PS (2021). Causes of blindness and vision impairment in 2020 and trends over 30 years, and prevalence of avoidable blindness in relation to VISION 2020: the Right to sight: an analysis for the global burden of disease study. Lancet Glob Health.

[2] Wong WL, Su X, Li X (2014). Global prevalence of age-related macular degeneration and disease burden projection for 2020 and 2040: a systematic review and meta-analysis. Lancet Glob Health.

[3] Flaxman SR, Bourne RRA, Resnikoff S (2017). Global causes of blindness and distance vision impairment 1990–2020: a systematic review and meta-analysis. Lancet Glob Health.

[4] Lindblad AS, Kassoff A, Kieval S (1999). The age-related eye disease study (AREDS): design implications AREDS report no. 1. Control Clin Trials.

[5] Ferris FL, Wilkinson CP, Bird A (2013). Clinical classification of age-related macular degeneration. Ophthalmology.

[6] Chakravarthy U, Peto T (2020). Current perspective on age-related macular degeneration. JAMA.

[7] Sunness JS, Gonzalez-Baron J, Applegate CA (1999). Enlargement of atrophy and visual acuity loss in the geographic atrophy form of age-related macular degeneration. Ophthalmology.

[8] García-Layana A, Cabrera-López F, García-Arumí J (2017). Early and intermediate age-related macular degeneration: update and clinical review. Clin Interv Aging.

[9] Cabral De Guimaraes TA, Daich Varela M, Georgiou M, Michaelides M (2021). Treatments for dry age-related macular degeneration: therapeutic avenues, clinical trials and future directions. Br J Ophthalmol.

[10] Midena E, Pilotto E (2017). Microperimetry in age: related macular degeneration. Eye.

[11] Molina-Martín A, Pérez-Cambrodí RJ, Piñero DP (2018). Current clinical application of microperimetry: a review. Seminars Ophthalmol.

[12] Pfau M, Jolly JK, Wu Z (2020). Fundus-controlled perimetry (microperimetry): application as outcome measure in clinical trials. Prog Retin Eye Res.

[13] Nebbioso M, Barbato A, Pescosolido N (2014). Scotopic microperimetry in the early diagnosis of age-related macular degeneration: preliminary study. Biomed Res Int.

[14] Corvi F, Pellegrini M, Belotti M (2019). Scotopic and fast mesopic microperimetry in eyes with drusen and reticular pseudodrusen. Retina.

[15] Crossland MD, Luong VA, Rubin GS, Fitzke FW (2011). Retinal specific measurement of dark-adapted visual function: validation of a modified microperimeter. BMC Ophthalmol.

[16] Steinberg JS, Fitzke FW, Fimmers R (2015). Scotopic and photopic microperimetry in patients with reticular drusen and age-related macular degeneration. JAMA Ophthalmol.

[17] Steinberg JS, Sassmannshausen M, Pfau M (2017). Evaluation of two systems for fundus-controlled scotopic and mesopic perimetry in eye with age-related macular degeneration. Trans Vis Sci Tech.

[18] Bowl W, Lorenz B, Jäger M, Friedburg C (2013). Improving detection of mild loss of retinal light increment sensitivity at the posterior pole with the Microperimeter MP1. Invest Ophthalmol Vis Sci.

[19] Microperimeter MP-3|Retina & Glaucoma | NIDEK CO.,LTD. https://www.nidek-intl.com/product/ophthaloptom/diagnostic/dia_retina/mp-3.html. Accessed 27 Apr 2021

[20] Pfau M, Lindner M, Fleckenstein M (2017). Test-retest reliability of scotopic and mesopic fundus-controlled perimetry using a modified MAIA (macular integrity assessment) in normal eyes. Ophthalmologica.

[21] Welker SG, Pfau M, Heinemann M (2018). Retest reliability of mesopic and dark-adapted microperimetry in patients with intermediate age-related macular degeneration and age-matched controls. Invest Ophthalmol Vis Sci.

[22] Pfau M, Lindner M, Gliem M (2018). Mesopic and dark-adapted two-color fundus-controlled perimetry in patients with cuticular, reticular, and soft drusen. Eye.

[23] Pondorfer SG, Heinemann M, Wintergerst MWM (2020). Detecting vision loss in intermediate agerelated macular degeneration: a comparison of visual function tests. PLoS ONE.

[24] Pfau M, Müller PL, von der Emde L (2020). Mesopic and dark-adapted two-color fundus-controlled perimetry in geographic atrophy secondary to age-related macular degeneration. Retina.

[25] Montesano G, Ometto G, Higgins BE (2020). Structure–function analysis in macular drusen with mesopic and scotopic microperimetry. Trans Vis Sci Tech.

[26] Sassmannshausen M, Steinberg JS, Fimmers R (2018). Structure-function analysis in patients with intermediate age-related macular degeneration. Invest Ophthalmol Vis Sci.

[27] von der Emde L, Pfau M, Thiele S (2019). Mesopic and dark-adapted two-color fundus-controlled perimetry in choroidal neovascularization secondary to age-related macular degeneration. Trans Vis Sci Tech.

[28] Munn Z, Peters MDJ, Stern C (2018). Systematic review or scoping review? Guidance for authors when choosing between a systematic or scoping review approach. BMC Med Res Methodol.

[29] Madheswaran G, Nasim P, SVe R, et al (2021) OSF Registries | Scotopic perimetry in age related macular degeneration: scoping review protocol. https://osf.io/zpqk6. Accessed 5 Mar 2021

[30] Arksey H, O’Malley L (2005). Scoping studies: towards a methodological framework. Int J Social Res Method Theory Pract.

[31] Levac D, Colquhoun H, O’Brien KK (2010). Scoping studies: advancing the methodology. Implement Sci.

[32] Peters MDJ, Marnie C, Tricco AC (2021). Updated methodological guidance for the conduct of scoping reviews. JBI Evidence Implementation.

[33] Mendeley Reference Manager. https://www.mendeley.com/reference-manager/library/all-references. Accessed 5 May 2021

[34] Tricco AC, Lillie E, Zarin W (2018). PRISMA extension for scoping reviews (PRISMA-ScR): checklist and explanation. Ann Intern Med.

[35] Cassels N (2017). Quality-of-life and clinical outcomes in age-related macular degeneration.

[36] Grewal MK (2021) Visual function in aging and age-related macular degeneration including subretinal drusenoid deposits. UCL (University College London)

[37] Steinberg JS, Sassmannshausen M, Fleckenstein M (2016). Correlation of partial outer retinal thickness with scotopic and mesopic fundus-controlled perimetry in patients with reticular drusen. Am J Ophthalmol.

[38] Sassmannshausen M, Pfau M, Thiele S (2020). Longitudinal analysis of structural and functional changes in presence of reticular pseudodrusen associated with age-related macular degeneration. Invest Ophthalmol Vis Sci.

[39] Sassmannshausen M, Zhou J, Pfau M (2021). Longitudinal analysis of retinal thickness and retinal function in eyes with large drusen secondary to intermediate age-related macular degeneration. Ophthalmol Retina.

[40] Chandramohan A, Stinnett SS, Petrowski JT (2016). Visual function measures in early and intermediate age-related macular degeneration. Retina.

[41] Forte R, Cennamo GG, De Crecchio G, Cennamo GG (2013). Microperimetry of subretinal drusenoid deposits. Ophthalmic Res.

[42] Querques G, Massamba N, Srour M (2014). Impact of reticular pseudodrusen on macular function. Retina.

[43] Pascolini D, Mariotti SP (2012). Global estimates of visual impairment: 2010. Br J Ophthalmol.

[44] Joachim NDL, Mitchell P, Kifley A, Jinwang J (2015). Incidence, progression, and associated risk factors of medium drusen in age-related macular degeneration findings from the 15-year follow-up of an Australian cohort. JAMA Ophthalmol.

[45] Chakravarthy U, Bailey CC, Scanlon PH (2020). Progression from early/intermediate to advanced forms of age-related macular degeneration in a large UK Cohort: rates and risk factors. Ophthalmol Retina.

[46] Murdoch IE, Morris SS, Cousens SN (1998). People and eyes: statistical approaches in ophthalmology. Br J Ophthalmol.

