# Supplementary material for: Role of microperimetry in evaluating disease progression in age-related macular degeneration: a scoping review
Source: Int Ophthalmol. 2022 Jan 7;42(6):1975–86. doi: 10.1007/s10792-021-02170-9 (PMC9156461; doi:10.1007/s10792-021-02170-9)
Supplement: Supplementary file 1 — Supplementary file1 (DOCX 15 KB) [file 10792_2021_2170_MOESM1_ESM.docx]

**Title:** Role of microperimetry in evaluating disease progression in age-related macular degeneration: a scoping review

**Authors:** Gopinath Madheswaran^1^, Pinaz Nasim^1^, Shonraj Ballae Ganeshrao^1,^ Rajiv Raman^2,^ Ramesh SVe^1^*

**Affiliations:**

*^1^Department of Optometry, Manipal College of Health Professions, Manipal Academy of Higher Education, Manipal, Karnataka, India.*

*^2^Shri Bhagwan Mahavir Vitreoretinal Services, Sankara Nethralaya, Chennai, Tamilnadu, India.*

***Corresponding author:**

Dr Ramesh SVe, Email: [ramesh.sve@manipal.edu](mailto:ramesh.sve@manipal.edu)

**Online Resource 2**

| **Study** | **Reasons for exclusion** | |
| --- | --- | --- |
|  | **Microperimetry procedural differences** | **Methodological differences** |
| Haimovici R et al., 2002 |  | Dark-adapted perimetry using modified Humphrey visual field |
| Roman A J et al., 2005 | Not measuring scotopic and mesopic microperimetry sensitivities |  |
| Kiser AK et al., 2006 |  | Dark adaptometry and dark-adapted Humphrey macular perimetry |
| Midena E et al., 2007 | Not measuring scotopic and mesopic microperimetry sensitivities |  |
| Meleth AD et al., 2011 | Not measuring scotopic and mesopic microperimetry sensitivities |  |
| Tepelus, T.C. et al., 2011 | Measured only mesopic microperimetry sensitivity |  |
| Crossland MD et al., 2011 | Measured only scotopic microperimetry sensitivity |  |
| Querques G et al., 2014 | Not measuring scotopic and mesopic microperimetry sensitivities |  |
| Wu Z et al., 2015 | Not measuring scotopic and mesopic microperimetry sensitivities |  |
| Wu Z et al., 2015 | Not measuring scotopic and mesopic microperimetry sensitivities |  |
| Liu H et al., 2015 | Not measuring scotopic and mesopic microperimetry sensitivities |  |
| Wu Z et al., 2015 | Not measuring scotopic and mesopic microperimetry sensitivities |  |
| Loughman J et al., 2015 | Not measuring scotopic and mesopic microperimetry sensitivities |  |
| Fraser RG et al., 2016 |  | Medmont Dark-Adapted Chromatic Perimeter |
| Wu Z et al., 2016 | Not measuring scotopic and mesopic microperimetry sensitivities |  |
| Takahashi A et al., 2016 | Not measuring scotopic and mesopic microperimetry sensitivities |  |
| Chandramohan A et al., 2016 | Not measuring scotopic and mesopic microperimetry sensitivities |  |
| Neely D et al., 2017 |  | Measuring rod mediated dark adaptation measurement using AdaptDx |
| Pfau M et al., 2017 | Measured only scotopic microperimetry sensitivity |  |
| Steinberg JS et al., 2017 | Not measuring scotopic and mesopic microperimetry sensitivities |  |
| Vujosevic S et al., 2017 | Measured only mesopic microperimetry sensitivity |  |
| Nguyen CT et al., 2018 |  | Medmont Dark-Adapted Chromatic Perimeter |
| Tan RS et al., 2018 |  | Medmont Dark-Adapted Chromatic Perimeter |
| Cocce KJ et al., 2018 |  | Dark adaptometry (AdaptDx) |
| McGuinness MB et al., 2020 |  | Medmont Dark-Adapted Chromatic Perimeter |
| Wu Z et al., 2021 | Not measuring scotopic and mesopic microperimetry sensitivities |  |
| Nassisi M et al., 2021 | Measured only scotopic microperimetry sensitivity |  |
